# Curriculum „Trachealkanülenmanagement in der Dysphagietherapie“

**DOI:** 10.1007/s00115-023-01598-x

**Published:** 2024-01-26

**Authors:** C. Ledl, U. Frank, R. Dziewas, B. Arnold, N. Bähre, C. S. Betz, S. Braune, T. Deitmer, P. Diesener, A. S. Fischer, S. Hamzic, G. Iberl, J. Konradi, J. Löhler, T. Platz, C. Rohlfes, M. Westhoff, S. Winkler, R. Wirth, S. Graf

**Affiliations:** 1 Deutsche interdisziplinäre Gesellschaft für Dysphagie, München, Deutschland; 2 Schön Klinik Bad Aibling, Bad Aibling, Deutschland; 3 Swallowing Research Lab, Universität Potsdam, Potsdam, Deutschland; 4 Deutsche Gesellschaft für Neurologie, Berlin, Deutschland; 5 Deutsche Gesellschaft für NeuroIntensiv- und Notfallmedizin, Jena, Deutschland; 6 Klinik für Neurologie und neurologische Frührehabilitation, Klinikum Osnabrück, Osnabrück, Deutschland; 7 Deutscher Berufsverband für Phoniatrie und Pädaudiologie, Berlin, Deutschland; 8 Deutsche Interdisziplinäre Gesellschaft für Außerklinische Beatmung, Freiburg, Deutschland; 9 Deutsche Gesellschaft für Hals-Nasen-Ohren Heilkunde, Kopf- und Hals-Chirurgie, Bonn, Deutschland; 10 Klinik und Poliklinik für HNO-Heilkunde, Universitätsklinikum Hamburg-Eppendorf, Hamburg, Deutschland; 11 Deutsche Gesellschaft für Pneumologie und Beatmungsmedizin, Berlin, Deutschland; 12 St. Franziskus-Hospital Münster, Münster, Deutschland; 13 Dysphagie-Netzwerk-Südwest e. V., Überlingen, Deutschland; 14 Dysphagie- und Kanülensprechstunde Hegau-Jugendwerk Gailingen, Gailingen, Deutschland; 15 Rehaklinik Zihlschlacht, Zihlschlacht-Sitterdorf, Schweiz; 16 Juristische Fakultät, Forschungsstelle Medizinrecht, Ludwig-Maximilians-Universität, München, Deutschland; 17 Deutscher Bundesverband für akademische Sprachtherapie und Logopädie, Moers, Deutschland; 18 Universitätsklinikum Gießen und Marburg, Campus Gießen, Neurologische Klinik, Justus-Liebig-Universität, Gießen, Deutschland; 19 Institut für Physikalische Therapie, Prävention und Rehabilitation, Universitätsmedizin der Johannes-Gutenberg-Universität Mainz, Mainz, Deutschland; 20 Deutscher Berufsverband der HNO-Ärzte, Neumünster, Deutschland; 21 Deutsche Gesellschaft für Neurorehabilitation, Berlin, Deutschland; 22 Institut für Neurorehabilitation und Evidenzbasierung, An-Institut der Universität Greifswald, BDH-Klinik Greifswald, Greifswald, Deutschland; 23 AG Neurorehabilitation, Universitätsmedizin Greifswald, Greifswald, Deutschland; 24 BDH-Klinik Hessisch Oldendorf, Hessisch Oldendorf, Deutschland; 25 Zentrum für Pneumologie und Thoraxchirurgie, Lungenklinik Hemer, Hemer, Deutschland; 26 Deutscher Bundesverband für Logopädie, Frechen, Deutschland; 27 Deutsche Gesellschaft für Geriatrie, Berlin, Deutschland; 28 Klinik für Altersmedizin, Marien Hospital Herne, Universitätsklinikum der Ruhr-Universität Bochum, Bochum, Deutschland; 29 Deutsche Gesellschaft für Phoniatrie und Pädaudiologie, Göttingen, Deutschland; 30 Universitätsklinik für Hör‑, Stimm- und Sprachstörungen, Medizinische Universität Innsbruck, Innsbruck, Österreich

**Keywords:** Dysphagie, Trachealkanüle, Dekanülierung, Weaning, Tracheotomie, Dysphagia, Tracheal cannula, Decannulation, Weaning, Tracheostomy

## Abstract

Die Anzahl tracheotomierter dysphagischer PatientInnen im klinischen und außerklinisch-ambulanten Setting und der damit einhergehende Behandlungsbedarf steigen kontinuierlich. Die Neufassung der Richtlinie des Gemeinsamen Bundesausschusses über die Verordnung außerklinischer Intensivpflege (AKI) sieht zudem vor, dass PatientInnen in der AKI regelmäßig evaluiert werden, um Therapiepotenzial nach stationärer Entlassung zu erkennen und zu fördern. Eine besondere Rolle nimmt dabei die Dysphagietherapie ein, da ohne Besserung schwerer Dysphagien kaum die Möglichkeit einer Dekanülierung besteht. Tracheotomierte dysphagische PatientInnen werden von LogopädInnen und akademischen SprachtherapeutInnen behandelt. Inhalte zum Trachealkanülenmanagement (TKM) finden sich jedoch nicht obligatorisch in den sprachtherapeutisch-logopädischen Ausbildungs-Curricula, sodass Weiterbildungsbedarf im therapeutischen Umgang mit Trachealkanülen besteht und Behandlungsstandards gesichert werden müssen. Daher wurde von der Deutschen interdisziplinären Gesellschaft für Dysphagie (DGD) in Kooperation mit den beteiligten therapeutischen und medizinischen Fachgesellschaften ein Curriculum zum TKM entwickelt. Dieses soll Basis für das inhaltliche Vorgehen im TKM sein und als Qualifikationsnachweis der TherapeutInnen im Rahmen der Delegation ärztlicher Leistungen dienen. Ziele des TKM-Curriculums sind die Definition theoretischer und praktischer Weiterbildungsinhalte, die Befähigung zur Durchführung des TKM nach aktuellen Standards sowie die Qualitätssicherung. Das Curriculum definiert zwei Qualifikationsstufen (AnwenderIn und AusbilderIn), Eingangsvoraussetzungen, curriculare Inhalte, Prüfungs- und Qualifikationskriterien sowie Übergangsregelungen für bereits im TKM erfahrene TherapeutInnen.

## Abstract (en)

The number of tracheotomized patients with dysphagia and their need for treatment are continuously increasing in clinical and community settings. The revised version of the directive on home care and community-based intensive care of the Federal Joint Committee (G-BA) requires that tracheotomized patients are regularly evaluated with the aim of identifying and promoting the therapeutic potential after hospital discharge. Dysphagia treatment plays a crucial role as without improvement of severe dysphagia there is practically no possibility for decannulation. Tracheotomized patients with dysphagia are treated by speech and language therapists (SLT); however, the contents of tracheostomy management (TM) are not obligatory in the speech and language therapeutic training curricula, so that there is a need for further education and treatment standards must be secured. Therefore, the German Interdisciplinary Society for Dysphagia (DGD) in cooperation with the participating German medical and therapeutic societies developed a postgraduate curriculum for TM. This should serve as the basis for contents in TM and qualification of therapists within the framework of the delegation of medical services. The goals of the TM curriculum are the definition of theoretical and practical contents of TM, the qualification to perform TM according to current standards of care and quality assurance. The curriculum defines two qualification levels (user and trainer), entry requirements, curricular contents, examination and qualification criteria as well as transitional regulations for SLTs already experienced in TM.

## 1. Einleitung

Die Behandlung tracheotomierter PatientInnen mit Dysphagie ist Tätigkeitsfeld von LogopädInnen und akademischen SprachtherapeutInnen (LogaS) im intensiv- und akutmedizinischen, rehabilitativen und außerklinischen Setting. Die Dysphagietherapie nimmt dabei einen zentralen Stellenwert ein, da Dekanülierungen häufig an dysphagiebedingten Komplikationen, insbesondere rezidivierenden Infektionen der unteren Atemwege, scheitern [1, 2]. Außerdem werden durch therapeutisches Trachealkanülenmanagement Vorteile im Beatmungsweaning beschrieben, z. B. verbesserter Gasaustausch, verringerte Aufenthaltsdauer auf Intensivstation [3, 4].

PatientInnen in neurologischen Weaningzentren zeigen neben der Beatmungspflichtigkeit eine sehr hohe Dysphagierate. In einer deutschen multizentrischen Studie hatten 87 % der zum Weaning aufgenommenen PatientInnen eine Schluckstörung, die Entwöhnung von der Beatmung gelang bei 65 % der PatientInnen, die Dekanülierungsrate lag hingegen nur bei 54 % [5, 6].

In der außerklinischen Intensivpflege (AKI) wird aufgrund des medizinisch-technischen Fortschritts ein kontinuierlicher Anstieg der PatientInnenzahlen beobachtet [7]. Dazu ist von einer großen Anzahl von PatientInnen in der AKI auszugehen, die aufgrund einer absaugpflichtigen Dysphagie tracheotomiert sind [8]. Der logopädisch-sprachtherapeutische Versorgungsanspruch dieses PatientInnenkollektivs wird durch die Neufassung der Richtlinie des Gemeinsamen Bundesausschusses über die Verordnung außerklinischer Intensivpflege (Außerklinische Intensivpflege-Richtlinie/AKI-RL, BAnz AT 17.03.2022 B2. https://www.g-ba.de/beschluesse/5142/. Zugriff: 17.10.2023) ansteigen. Die Richtlinie sieht vor, PatientInnen in der AKI regelmäßig zu evaluieren, um Therapiepotenziale auch nach stationärer Entlassung systematisch zu erkennen und zu fördern.

Zur Gewährleistung der PatientInnenversorgung und zur Verbesserung deren funktioneller Selbständigkeit und Teilhabe ist eine standardisierte und evidenzbasierte Qualifizierung der LogaS im TK-Management (TKM) erforderlich. Aktuell finden sich Inhalte zum TKM jedoch nicht obligatorisch in den Curricula der logopädischen und akademisch-sprachtherapeutischen Ausbildung. Die Notwendigkeit einer standardisierten Ausbildung zeigt eine multizentrische Studienserie in Großbritannien, in der drei Faktoren identifiziert wurden, die mit tracheotomiebedingten Risikosituationen und einer erhöhten Mortalität von TK-PatientInnen assoziiert sind ([9, 10], vgl. auch [11–13]):

1. mangelndes Wissen und fehlende Kompetenz der am TKM beteiligten Berufsgruppen,
2. unzureichende Kommunikation im multidisziplinären Team,
3. inkonsistente, nichtstandardisierte Reaktionen auf Notfallsituationen.

Im vorliegenden Artikel wird eine standardisierte und durch die beteiligten Fachdisziplinen konsentierte curriculare Weiterbildung für LogaS vorgestellt. Damit wird ein Qualifikationsnachweis geschaffen, der dazu beitragen wird, den medikolegalen Rahmen zur Absicherung der delegierenden ÄrztInnen und der delegationsnehmenden TherapeutInnen zu formulieren.

*Zielsetzungen des TKM-Curriculums sind:*

1. Definition der theoretischen und praktischen Anforderungen der Aus- und Weiterbildung im TKM,
2. Sicherung der Behandlungsqualität für tracheotomierte PatientInnen in der klinischen und außerklinischen Versorgung durch Etablierung evidenzbasierter Qualitätsstandards,
3. Transparenz in der interprofessionellen Kommunikation,
4. formale Qualifikation der TherapeutInnen als Basis der Delegation TKM-bezogener Tätigkeiten.

Anwendungsgebiete des Curriculums sind das ambulante und stationäre TKM im Kontext der Dysphagietherapie in Neurologie und neurologischer Rehabilitation, Hals-Nasen-Ohrenheilkunde, Phoniatrie, Intensivmedizin, Pulmologie/Pneumologie, Pädiatrie und Geriatrie.

Das hier vorgestellte TKM-Curriculum wurde von der Deutschen interdisziplinären Gesellschaft für Dysphagie (DGD) in Zusammenarbeit mit den folgenden Fachgesellschaften entwickelt und konsentiert:

dbl: Deutscher Bundesverband für Logopädie
dbs: Deutscher Bundesverband für akademische Sprachtherapie und Logopädie
DGD: Deutsche interdisziplinäre Gesellschaft für Dysphagie
DGG: Deutsche Gesellschaft für Geriatrie
DGHNO-KHC: Deutsche Gesellschaft für Hals-Nasen-Ohren-Heilkunde, Kopf- und Hals-Chirurgie
BV-HNO: Deutscher Berufsverband der HNO-Ärzte
DGN: Deutsche Gesellschaft für Neurologie
DGNI: Deutsche Gesellschaft für NeuroIntensiv- und Notfallmedizin
DGNR: Deutsche Gesellschaft für Neurorehabilitation
DGP: Deutsche Gesellschaft für Pneumologie und Beatmungsmedizin
DGPP: Deutsche Gesellschaft für Phoniatrie und Pädaudiologie
DBVPP: Deutscher Berufsverband für Phoniatrie und Pädaudiologie
DIGAB: Deutsche interdisziplinäre Gesellschaft für außerklinische Beatmung

Das TKM-Curriculum orientiert sich inhaltlich an den Empfehlungen der Leitlinien der Deutschen Gesellschaft für Neurologie (DGN; [14]), der Deutschen Gesellschaft für Pneumologie und Beatmungsmedizin (DGP; [2, 15]), der Deutschen Gesellschaft für Neurorehabilitation (DGNR; [16]), der DGHNO-KHC [17] und der Deutschen Gesellschaft für Phoniatrie und Pädaudiologie (DGPP; [18]) sowie an den Guidelines des Royal College of Speech Language Therapists, UK (RCSLT; [19]) und der Global Tracheostomy Collaborative (GTC; [20]).

## 2. TKM-Curriculum: Qualifikationsstufen

Das TKM-Curriculum umfasst zwei Qualifikationsstufen:

- TKM-Zertifikat (TKM-AnwenderInnen),
- TKM-Ausbildungs-Zertifikat (TKM-AusbilderInnen).

*Zerifizierte TKM-AnwenderInnen* sind LogaS, die die diagnostischen und therapeutischen Verfahren des TKM im klinischen, außerklinischen und/oder ambulanten Setting durchführen. Der Tätigkeitsbereich ergibt sich aus den Inhalten des Basisseminars (siehe 3.2).

*TKM-AusbilderInnen* sind zertifizierte TKM-AnwenderInnen, die zusätzlich berechtigt sind, TKM-Basisseminare und direkte und indirekte Behandlungssupervisionen durchzuführen und Prüfungen für das TKM-Zertifikat abzunehmen. TKM-AusbilderInnen müssen über umfassende klinische Kenntnisse und Fertigkeiten im TKM und über didaktische Kompetenzen verfügen. Sie sind in der Lage, auch komplexe Fälle selbständig zu evaluieren und geeignete Vorgehensweisen im multidisziplinären Team zu initiieren, durchzuführen und anzuleiten (siehe 4).

## 3. TKM-Zertifikat

### 3.1. Eingangsvoraussetzungen und Ausbildungsabschnitte

Für die Zulassung zur Weiterbildung im TKM-Zertifikat müssen die in Tab. 1 definierten Eingangsvoraussetzungen erfüllt sein. Die Ausbildung zum Erwerb des TKM-Zertifikats gliedert sich in drei Abschnitte:

1. *TKM-Basisseminar* (siehe 3.2): Vermittlung von Grundlagenkenntnissen und praktischen Handlungskompetenzen mit theoretischer und praktischer Prüfung,
2. *Supervisions- und Behandlungsphase* (siehe 3.3): TKM mit direkter und indirekter Supervision durch TKM-AusbilderInnen,
3. *Fallberichte* (siehe 3.4): Erstellen von 2 Fallberichten aus der Supervisionsphase oder analoge Leistungen (siehe 3.4),
4. *Abschlussprüfung* (siehe 3.5).

**Table 1:** 

Abschnitt	Erläuterung
*Eingangsvoraussetzungen*	Berufsabschluss „LogopädIn“ oder „akademische(r) SprachtherapeutIn“
	Mind. 2‑jährige spezifische berufspraktische Tätigkeit mit mindestens 20 behandelten DysphagiepatientInnen
	Mind. 30 h Aus- oder Fortbildung im Bereich Dysphagiemanagement
*Basisseminar*	30 UE
	Theoretische und praktische Prüfung
*Supervisions- und Behandlungsphase*	20 TE TKM direkte Supervision
	40 TE TKM indirekte Supervision
*Fallberichte*	2 Fallberichte oder analoge Leistungen (siehe 3.4)
*Abschlussprüfung*	Prüfungsgespräch (30 min)

*UE* Unterrichtseinheiten (45 min), *TE* Therapieeinheiten, *TKM* Trachealkanülenmanagement

### 3.2. TKM-Basisseminar: Erwerb von Grundlagenkenntnissen und praktischen Handlungskompetenzen

Die fachlichen Kompetenzen zum Erwerb des TKM-Zertifikats werden in einem Basisseminar mit einem Umfang von 30 Unterrichtseinheiten (1 UE = 45 min) vermittelt. Die in Tab. 2 aufgeführten Inhalte sind obligatorisch. Die zu erwerbenden praktischen Handlungskompetenzen werden im Basisseminar in kleinen Arbeitsgruppen mit maximal 5 TeilnehmerInnen pro TKM-AusbilderIn vermittelt und geprüft. Jeder Arbeitsgruppe müssen ein Trainingsdummy, ein Absauggerät, verschiedene TK-Modelle und ausreichend Verbrauchsmaterial zur Verfügung stehen.

**Table 2:** 

**Basisthemen**	**Grundlagen, Erwerb von Kenntnissen (12** **UE)**	**30** **UE**^**a**^	**ReferentInnen**^**b**^
*1. Tracheotomie*	Indikationen, Kontraindikationen bzw. Alternativen, Zeitpunkt und Arten der Tracheotomie	0,5	FÄ LogaS
	Implikationen zu Stabilität des Tracheostomas		
	Komplikationen der Tracheotomie		
	Auswirkungen der Tracheotomie auf Atemtiefe (Totraumverkleinerung)		
	Luftstromführung, Geruch, Geschmack, Husten, Phonation und Kommunikation		
*2. Trachealkanülen (TK)*	Indikationen, Komponenten und Varianten: Cuff, einlumige Kanüle und Kanüle mit Innenseele, Techniken der Kanülenfixierung, Fenestrierung, subglottische Absaugung, Vocal-Aid-Funktion, Beatmungskanülen, Platzhalter, Stomapflaster	1,5	FÄ LogaS Pfl-ITS
	Kriterien zur TK-Auswahl: Funktion, Größe, Außen- und Innendurchmesser, Länge, Material, Krümmungswinkel, Lage der Fenestrierung, Länge des Tracheostomakanals, Erkrankungsstadium		
	Bestimmung des Luftwegs mit unterschiedlichen TK-Varianten		
	Cuff-Blockung, Blockungsarten (Nieder‑, Hochdruck, Foam, Lanz), Cuff-Druck-Kontrolle, Entblockung, Wechsel der Innenkanülen, Ventile und Verschlusskappen		
*3. Absaugen*	Indikationsstellung	0,5	FÄ LogaS Pfl-ITS
	Vorbereitung und Material		
	Geschlossene Absaugsysteme		
	Transnasales, innerkanüläres, endotracheales Absaugen		
	Risiko- und Komplikationsmanagement, persönliche Schutzausrüstung		
*4. Pflege von Tracheostoma (TS) und TK*	TK-Reinigung, TS-Pflege	0,5	FÄ LogaS Pfl-ITS
	Tracheostomakompressen		
	Fixiertechniken und Prophylaxe von Stomaweitung u. a. Komplikationen		
	Befeuchtung und Entzündungsprophylaxe		
	TS-Komplikationen: Entzündungen, Stenosen, Granulationen, Blutungen, Ulzerationen		
*5. Trachealkanülenwechsel (TKW)*	Wechselintervalle und Indikationsstellungen	0,5	FÄ LogaS Pfl-ITS
	Material, Vorgehen, Risiken		
	TKW mit/ohne Führungsdraht		
	Möglichkeiten der TK-Lagekontrolle		
*6. TK-assoziierte Komplikationen*	Cuff-assoziierte Komplikationen	1,0	FÄ LogaS
	Fehllagen der TK (z. B. verdeckte Fenestrierung, tracheale und stomatale Druckläsionen)		
	Obstruktionen		
	Luftverlust parastomal und Abdichttechniken		
	Stridor, Trachealstenosen, Tracheomalazie, Blutungen, Hautemphysem		
	Diagnostik und Management TK-assoziierter Komplikationen		
*7. Notfallsituationen*	Monitoring	0,5	FÄ LogaS Pfl-ITS
	Kenntnis gestaffelter Notfallschemata		
	Notfallinterventionen im Kontext der TKM-Dysphagiebehandlung		
*8. Klinische und instrumentelle Diagnostik im TKM*	Screeningverfahren und klinische Schluckuntersuchung: Zeitpunkt und Vorgehen	2,0	LogaS FÄ
	Aspirationsdiagnostik/Färbetests: Indikation, Reliabilität, Durchführung		
	Instrumentelle TKM-Diagnostik: endoskopische (FEES) und radiologische (VFSS) Diagnostik, TK-Inspektion, Tracheoskopie und Bronchoskopie		
	Diagnostik der Hustenfunktion: quantitative und qualitative Methoden		
*9. Therapeutische Interventionen*	Kriterien und Kontraindikationen für Entblocken und sukzessives Okklusionstraining, Unterschiede Okklusion bei Verschlusskappen, exspiratorische Okklusion bei Sprechventilen	1,5	LogaS FÄ
	Indikation und therapeutische Intention der Luftstromführung und der verschiedenen Okklusionsformen, Effekte auf Sekret- und Speichelmanagement, Phonation und Hustenfunktion		
*10. Orale Ernährung*	Möglichkeiten und Risiken des oralen Kostaufbaus im Kontext des TKM	0,5	LogaS FÄ
*11. Speichel- und Sekretmanagement*	Dysphagietherapeutische Maßnahmen	0,5	LogaS FÄ Pfl-ITS
	Befeuchtung, Sekretmodulation, Sekretexpektoration		
	Medikamentöse Speichel- und Sekretreduktion		
*12. TK-Weaning*	Dysphagietherapeutische Maßnahmen zur Unterstützung der Dekanülierungsfähigkeit: Unterstützung von Speichelmanagement, Atemfunktion und Husteneffektivität	1,0	LogaS FÄ
	Dekanülierungspfade: spontane und gestufte Pfade		
	Indikation und Effekte des Downsizing, Anpassung des TK-Typs		
	Risikoabwägung und Absprachen im multidisziplinären Team		
*13. Dekanülierung*	Voraussetzungen und Kriterien für die Dekanülierbarkeit und Dekanülierungserfolg	1,0	LogaS FÄ
	Vorgehen bei der Dekanülierung		
	Dekanülierungshindernisse (z. B. persistierende schwere Dysphagie, insuffiziente Hustentriggerung und Hustenkraft, oropharyngeales und bronchiales Sekret, Beatmungsnotwendigkeit, Stenosen)		
	Schwierige Dekanülierungen (z. B. Platzhaltereinsatz)		
	Prozedere nach der Dekanülierung, Verschluss des Tracheostomas und Komplikationsprophylaxe		
	Techniken und Probleme der Platzhalteranlage, einschl. endoskopischer Kontrolle		
*14. Vorgehen bei nichtdekanülierbaren PatientInnen*	Gründe der Nichtdekanülierbarkeit (z. B. supra-/subglottische und glottale Stenosen, funktionelle Atemwegsstenosen, insuffizientes Sekretmanagement, insuffiziente Reinigungsfunktionen)	0,5	LogaS FÄ
	Epithelialisierung des Tracheostomas, TK-Anpassung		
	Möglichkeiten für Sprech‑, Ess- und Trinkfunktion		
	**TKM in der Pädiatrie (1** **UE)**		
*15. TK-Versorgung in der Pädiatrie*	TK-Auswahl bei Säuglingen und Kindern (ggf. Nichtanwendbarkeit der Tubusregel zur Sicherung eines ausreichenden TK-Innendurchmessers)	1,0	FÄ LogaS Pfl-ITS
	Komplikationen und Prävention: akzidentelle Dekanülierung, Druckulzera, Hypergranulation, subglottische und tracheale Stenosen, Infektionen		
	Familiäres Umfeld: Interaktion, Schulung		
*16. Dekanülierung*	Pädiatrische Dekanülierungspfade		
	Sprechventile und Gefahr von CO_2_-Retentionen		
	**Rechtliche und formale Aspekte (1** **UE)**		
*17. Rechtliche Aspekte*	Delegationsprinzip (materielle und formale Qualifikation, Pflichten der Delegierenden und der DelegationsnehmerInnen, insb. gegenseitige Information, Absprachen über das Vorgehen, Übernahmeverantwortung)	0,5	Juristen FÄ LogaS
	Medizinproduktegesetz		
*18. Formale Aspekte*	Multidisziplinäres Behandlungsteam: Aufgaben und Absprachen		
	Benefit und Implikationen standardisierter Prozesse, z. B. Absaugen, TK-Pflege, Dekanülierung		
	**TKM auf der Intensivstation (ICU; 4** **UE)**		
*19. ICU-spezifische Ätiologien*	Multifaktorielle Dysphagieursachen und Komplikationen, Postextubationsdysphagie, ICU Acquired Weakness	0,5	FÄ LogaS
	Veränderte Luftstromführung bei TK und Beatmung		
	Ösophagogastrale Motilitätsstörungen		
	Ventilatorinduzierte Zwerchfelldysfunktion		
	Akutes Lungenversagen (ARDS)		
*20. Beatmung und Intubation*	Anatomie und Physiologie der Atmung	1,5	FÄ Pfl-IST LogaS
	Pathophysiologie und Beatmungsindikationen		
	Funktion und Grundbegriffe maschineller Beatmung, invasive und nichtinvasive Beatmungstherapie, Beatmungsmodi		
	Endotracheale Intubation, Komplikationen der Intubation		
	Risiken und Langzeitfolgen der Beatmung		
*21. ICU-Besonderheiten des TKM*	Luftstromführung mit TK unter Beatmung	1,5	LogaS FÄ
	Above-Cuff-Vocalization		
	Leckage-Beatmung		
	Kriterien für Spontanatemversuche		
	Kriterien für die Cuff-Entblockung		
	Dekanülierungspfade und -kriterien		
	Physikalische und therapeutische Maßnahmen, mechanische In- und Exsufflatoren, NIV, High-Flow-Therapie, atmungstherapeutische Maßnahmen		
*22. Funktionelle Dysphagietherapie – ICU*	Lagerung und funktionelle Frühmobilisation	1	LogaS FÄ
	Orale Hygiene		
	Neuromodulatorische Therapieverfahren		
**Praxisthemen**	**Erwerb praktischer Handlungskompetenzen (11** **UE)**		
*Trachealkanülen-Handling*	Blocken/Entblocken der TK	2,0	LogaS FÄ Pfl-ITS
	Wechsel der Innenkanülen (fenestriert, nichtfenestriert)		
	Verwenden von HME-Filtern, Verschlusskappen und Sprechventilen		
	Sauerstoffzufuhr		
*Physiologische Luftstromführung*	Luftstromführung mit fenestrierter/nichtfenestrierter Innenkanüle/TK	1,5	LogaS FÄ
	Luftstromführung (Fingerverschluss, Sprechventil, Verschlusskappe)		
	Übungen zur Luftstromführung bei pädiatrischen PatientInnen		
	Übungen zur Luftstromführung und zur Above-Cuff-Vocalization bei beatmeten PatientInnen		
*Färbetests/Absaugen*	Absaugen transnasal, innerkanülär, endotracheal (am Übungsdummy), atraumatisches Absaugen	2,0	LogaS FÄ Pfl-ITS
	Mind. 10-mal je TeilnehmerIn/Modalität		
	Durchführung von Färbetests		
*Komplikationen und Notfallsituationen*	Training Ablauf von Notfallschemata für die unterschiedlichen Komplikationssituationen; Dekanülierung/Rekanülierung (Trachealkanülenwechsel mit und ohne Führungsdraht)	2,5	FÄ Pfl-ITS LogaS
	Mind. 10-mal je TeilnehmerIn		
*Übungen zur Diagnostik, Fallbesprechungen*	Verdeutlichung, Diskussion und Vertiefung der Inhalte durch Fallbeispiele	3	FÄ LogaS
	Entwicklung eines Diagnostik- und Behandlungsplans		
	Befundinterpretation nach klinischer oder instrumenteller Untersuchung bzw. Färbetest		

*FEES* flexible endoskopische Evaluation der Schluckfunktion, *VFSS* Videofluoroskopie des Schluckakts, *PLF* physiologische Luftstromführung, *NIV* nichtinvasive Beatmung, *ICU* Intensive Care Unit

^a^Basisseminar: 29 UE Unterricht + 1 UE Abschlussprüfung

^b^Akkreditierbare ReferentInnen: *FÄ* FachärztInnen (Chirurgie, HNO, Intensivmedizin, Innere Medizin, Neurologie, Phoniatrie und Pädaudiologie, Pneumologie) mit 2‑jähriger Erfahrung in der Behandlung tracheotomierter PatientInnen, *LogaS* LogopädInnen, akademische SprachtherapeutInnen mit TKM-Ausbildungszertifikat, *Pfl-ITS* Fachpflegekräfte für Intensivpflege und Anästhesie oder Pflegefachkräfte mit 2‑jähriger Intensiverfahrung

Lehrinhalte müssen dem jeweils aktuellen Stand der Forschung und den entsprechenden Leitlinien der beteiligten Fachgesellschaften entsprechen. Dabei können max. 8 Unterrichtseinheiten als Online-Format angeboten werden, die praktischen Übungen müssen als Präsenzformat konzipiert sein. Die Zusammensetzung des Lehrkörpers soll der Thematik entsprechend multiprofessionell und multidisziplinär erfolgen, sodass ärztliche und therapeutische Bereiche inhaltlich adäquat vertreten werden. Lehrinhalte sollen entsprechend ihrer Thematik prioritär den akkreditierungsfähigen Berufsgruppen zugeordnet werden. Akkreditierungsfähige Berufsgruppen für die einzelnen Themenbereiche und entsprechende Priorisierungen sind in Tab. 2 definiert.

### 3.3. Supervisions- und Behandlungsphase: TKM-Anwendung mit direkter und indirekter Supervision

Beide Qualifikationsstufen (TKM-Zertifikat, TKM-Ausbildungs-Zertifikat) beinhalten eine supervidierte TKM-Anwendungsphase. Dabei wird zwischen direkter und indirekter Supervision unterschieden.

#### TKM mit direkter Supervision

Direkte Supervision setzt die Präsenz und die unmittelbare Interventionsmöglichkeit einer TKM-Ausbilderin/eines TKM-Ausbilders voraus. Die Dauer der Behandlungseinheit beträgt mind. 30 min (zzgl. Vor- bzw. Nachbesprechung). Die supervidierten Behandlungen dienen der Vertiefung der im Basisseminar erlernten Kompetenzen, die dadurch am konkreten Fall weiter praktisch gefestigt werden.

#### TKM mit indirekter Supervision

Nach der direkten Supervisionsphase folgen indirekt supervidierte Therapieeinheiten im TKM selbständig ohne Präsenz der Supervisorin/des Supervisors, jedoch muss eine fallbezogene Besprechung der Behandlungen erfolgen. Die indirekt supervidierten TKM-Einheiten dienen der Festigung der fachlichen Kompetenzen. Die AnwenderInnen sollen dadurch in die Lage versetzt werden, klinische Befunde im TKM selbständig zu erheben, im Kontext des Gesamtbildes zu interpretieren und geeignete therapeutische Maßnahmen eigenständig auszuwählen und durchzuführen. Die Auswahl der PatientInnen zur Durchführung der indirekten Supervision erfolgt nach Rücksprache mit der Supervisorin/dem Supervisor oder der betreuenden Ärztin/dem betreuenden Arzt.

#### Anforderungen im TKM-Zertifikat

Zum Erwerb des TKM-Zertifikats werden nach erfolgreichem Bestehen des Basisseminars mindestens 20 TKM-Behandlungseinheiten mit direkter Supervision und weitere 40 TKM-Behandlungseinheiten mit indirekter Supervision durchgeführt, hierbei müssen jeweils mindestens 3 unterschiedliche PatientInnen behandelt werden.

### 3.4. Fallberichte

Nach der Supervisions- und Behandlungsphase werden 2 Fallberichte auf Grundlage der Erfahrungen aus der Supervisions- und Behandlungsphase erstellt. Struktur und Inhalt der Fallberichte sollen sich an den CARE-Guidelines (CAse REport Reporting Guidelines [21]) orientieren und folgende Aspekte berücksichtigen:

- Informationen zu PatientInnen, klinischem Setting und Krankheitsbild,
- klinische Befunde, verwendete Diagnostikmethoden, Interpretation und Therapieableitung,
- therapeutische Interventionen: Art und Durchführung, Behandlungsdauer und -verlauf, weitere relevante Interventionen,
- ggf. Nachuntersuchungen,
- Diskussion, Reflexion und Schlussfolgerungen,
- PatientInnenperspektive.

Entsprechende Vorlagen zur Erstellung der Falldarstellungen werden zur Verfügung gestellt (www.dg-dysphagie.de). Je ein Fallbericht kann durch eine qualifizierte Publikation/Vortragstätigkeit zum TKM ersetzt werden, d. h. ein begutachteter Fachartikel in einem anerkannten Fachjournal, Buchkapitel oder Buchpublikation oder ein eingereichter oder eingeladener Vortrag bei einem einschlägigen Fachkongress.

### 3.5. Prüfungen im TKM-Zertifikat

Die Prüfung der praktischen Handlungskompetenzen im Basisseminar erfolgt seminarbegleitend während bzw. direkt im Anschluss an die praktischen Übungen im Basisseminar. Die TKM-AusbilderInnen prüfen, ob zu den in Tab. 2 aufgeführten Praxisthemen ausreichende praktische Handlungskompetenzen vorliegen, um in die Supervisions- und Behandlungsphase überzugehen.

Das TKM-Basisseminar schließt mit einer theoretischen und praktischen Prüfung ab (Tab. 3). Dabei müssen die in Tab. 2 aufgeführten Basisthemen obligatorisch geprüft werden. Die theoretische Prüfung soll im Multiple-Choice-Format mit mindestens 25 Fragen erfolgen, mindestens 60 % der Fragen müssen korrekt beantwortet werden (Tab. 3).

**Table 3:** 

*Theoretische Prüfung (Basisseminar)*	*Mind 25 Fragen.* dabei mindestens 1 Frage aus jedem der 22 Basisthemen (Tab. 2)
*Praktische Prüfung (Basisseminar)*	*Mind. 45* *min, Prüfungsbereiche:* Trachealkanülen-Handling Physiologische Luftstromführung und Okklusionstraining Färbetests/Absaugen Komplikationen und Notfallinterventionen
*Fallberichte*	*2 Fallberichte* oder analoge Leistungen (siehe 3.4)
*Abschlussprüfung*	*Prüfungsgespräch (mind. 30* *min):* Erläuterung und vertiefende Fragen zu den eingereichten Fallberichten, Grundlagenkenntnissen und praktischen Handlungskompetenzen, 2 PrüferInnen bzw. 1 PrüferIn und 1 BeisitzerIn^a^; SupervisorIn und PrüferIn dürfen nicht identisch sein

^a^BeisitzerInnen müssen das TKM-Zertifikat haben

Vor der Zulassung zur Abschlussprüfung müssen die direkt und indirekt supervidierten Praxiseinheiten absolviert und die Fallberichte (oder Nachweis analoger Leistungen, siehe 3.4) beim Prüfungskomitee eingereicht werden (siehe 7).

## 4. TKM-Ausbildungs-Zertifikat

Das TKM-Ausbildungs-Zertifikat kann durch Nachweis vertiefter Kenntnisse und praktischer Handlungskompetenzen, einer weiteren Supervisions- und Behandlungsphase sowie durch erfolgreiches Bestehen einer fachlich-didaktischen Prüfung erworben werden.

### 4.1. Eingangsvoraussetzungen und Ausbildungsabschnitte

Für die Prüfungszulassung zum TKM-Ausbildungs-Zertifikat müssen die in Tab. 4 definierten Eingangsvoraussetzungen nachgewiesen werden. Mit diesen Qualifikationsmerkmalen belegen TKM-AusbilderInnen, dass sie in komplexen klinischen Behandlungssituationen fachgerecht agieren können. Komplexe klinische Verläufe im TKM erfordern die Beteiligung und Koordination mehrerer interdisziplinärer Fachgruppen und sind gegeben beim Vorliegen spezifischer bzw. seltener Dekanülierungshindernisse und Komplikationen (z. B. pulmonale Komplikationen, Atemwegsstenosen). Die Komplikationen und die sich daraus ergebenden Vorgehensweisen müssen in den Fallberichten transparent und nachvollziehbar geschildert und sachgerecht reflektiert werden (positive und negative Aspekte). Darüber hinaus gelten die oben beschriebenen Vorgaben zu Struktur, Inhalt und Ersatz durch eine Publikation/Vortragstätigkeit (siehe 3.4).

**Table 4:** 

Abschnitt	Erläuterung
*Eingangsvoraussetzungen*	TKM-Zertifikat
	Mind. 4‑jährige spezifisch berufspraktische Tätigkeit (Dysphagietherapie)
	Mindestens 2‑jähriger Tätigkeitsschwerpunkt im Bereich TKM nach Erwerb des TKM-Zertifikats mit mind. 40 behandelten tracheotomierten DysphagiepatientInnen
	Nachweis von insgesamt 30 direkt supervidierten und insgesamt 100 indirekt supervidierten TKM-TE (mind. 5 unterschiedliche PatientInnen)^a^
	4 Fallberichte^a^ (davon mind. ein komplexer klinischer Verlauf) oder analoge Leistungen
*Prüfung*	*Prüfungsgespräch (mind. 60* *min), Themenbereiche:*
	Erläuterungen zu den eingereichten Fallberichten
	Kenntnisse der empirischen Basis, klinischer Transfer
	Problemstellungen und Lösungen in komplexen klinischen Situationen, bei pädiatrischen TK-PatientInnen und im ICU-Setting
	Einschätzung und Reflexion unterschiedlicher Vorgehensweisen und Behandlungspfade im TKM
	Erfahrungen, Hürden und Motivation in der Ausbildung von TKM-TherapeutInnen
	2 PrüferInnen bzw. 1 PrüferIn und 1 BeisitzerIn^b^

*TKM* Trachealkanülenmanagement, *TE* Therapieeinheiten

^a^Die supervidierten Behandlungseinheiten und Fallberichte aus dem TKM-Zertifikat werden angerechnet

^b^BeisitzerInnen müssen das TKM-Zertifikat haben

### 4.2. Prüfungen im TKM-Ausbildungs-Zertifikat

Beim Vorliegen der Eingangsvoraussetzungen (siehe Tab. 4) wird das TKM-Ausbildungs-Zertifikat durch Bestehen einer mündlichen Abschlussprüfung erworben. Die Prüfung dauert mindestens 60 min und umfasst vertiefende Fragen zu den eingereichten Fallberichten, Fragen zu inhaltlichen Kenntnissen und Transferwissen zu mindestens 3 Basisthemen des TKM-Basisseminars, Fragen zur Kompetenz in Wissensvermittlung und -darstellung sowie Fragen zur Abwägung komplexer Themen (siehe Tab. 3).

## 5. Prüfungsberechtigung zum TKM-Ausbildungs-Zertifikat

Zertifizierte TKM-AusbilderInnen können eine Prüfungsberechtigung für Prüfungen im TKM-Ausbildungs-Zertifikat erwerben und damit eine aktive Rolle übernehmen bei Prüfungen zum TKM-Ausbildungs-Zertifikat sowie im kontinuierlichen Monitoring und der Weiterentwicklung des TKM-Curriculums.

Die Prüfungsberechtigung zum TKM-Ausbildungs-Zertifikat setzt eine mindestens 2‑jährige aktive Tätigkeit als TKM-AusbilderIn voraus mit Nachweis von mindestens 20 behandelten komplexen TK-PatientInnen (siehe 4.1). Außerdem müssen qualitätssichernde TKM-Routinen und Standards im eigenen Arbeitssetting implementiert worden sein und nachgewiesen werden. Während der Tätigkeit als TKM-AusbilderIn muss eine Organisations- und ReferentInnentätigkeit in mindestens 2 TKM-Basisseminaren und die Beteiligung an Ausbildung und Prüfung von mindestens 5 TKM-AnwenderInnen (TKM-Zertifikat) erfolgt sein.

Die Prüfungsberechtigung zum TKM-Ausbildungs-Zertifikat wird abschließend erworben nach einem erfolgreichen Prüfungsgespräch (mind. 60 min), das durch 2 Mitglieder des TKM-Prüfungskomitees geführt wird. In der Phase der Übergangsregelung (siehe 6) stellt das Konsentierungskomitee die PrüferInnen für das TKM-Ausbildungs-Zertifikat.

## 6. Übergangsregelungen

Im Rahmen einer Übergangsregelung können bereits im TKM tätige LogaS das TKM-Zertifikat und das TKM-Ausbildungs-Zertifikat innerhalb eines Jahres nach Veröffentlichung des TKM-Curriculums beantragen. Dabei sind die in Tab. 5 angegebenen Nachweise zu erbringen.

**Table 5:** 

Qualifikationsstufe	Erläuterung
*TKM-Zertifikat*	Mind. 2‑jährige berufspraktische Tätigkeit mit mind. 2 Jahren Erfahrung im TKM nach dem Examen
	Nachweis von mindestens 20 behandelten tracheotomierten PatientInnen mit mind. 500 TKM-TE (mind. 30 min; siehe 7)
	Nachweis von mind. 30 h Aus- oder Weiterbildung im Dysphagiemanagement
	2 Fallberichte oder analoge Leistungen (siehe 4.3)
	Theoretische und praktische Prüfung (mind. 20 min): Kenntnisse, Transferwissen und praktische Handlungskompetenzen im Bereich TK-Handling und Okklusionstraining, Absaugen, Komplikationen und Notfallinterventionen (Tab. 2)
*TKM-Ausbildungs-Zertifikat*	Mind. 4‑jährige berufspraktische Schwerpunkttätigkeit im TKM
	Nachweis von mind. 30 h Aus- oder Weiterbildung im Dysphagiemanagement
	Nachweis von mindestens 60 behandelten tracheotomierten PatientInnen mit mind. 750 TKM-TE (mind. 30 min; siehe 7)
	Einweisung und praktische Anleitung in das TKM von mind. 3 LogaS; alternativ können je 20 UE (à 45 min) Lehr- oder Ausbildungstätigkeit im TKM anerkannt werden
	2 Fallberichte, davon mind. 1 komplexer Fall bzw. analoge Leistungen (siehe 3.4)
	Theoretische und praktische Prüfung (mind. 60 min): fachliche Kompetenzen und Transferwissen im Bereich TK-Handling und Okklusionstraining, Absaugen, Komplikationen und Notfallmanagement, Kompetenz in Wissensvermittlung, Darstellung und Abwägung komplexer Themen

*TKM* Trachealkanülenmangement, *TE* Therapieeinheiten, *LogaS* LogopädInnen und akademische SprachtherapeutInnen

## 7. Akkreditierung curricularer TKM-Basisseminare, Prüfung der Eingangsvoraussetzungen, Prüfungszulassung und Beantragung der TKM-Zertifikate

Initiatorin, federführende Fachgesellschaft und Zertifikatseignerin ist die DGD. Das Prüfungskomittee besteht aus Mitgliedern des Konsentierungsgremiums und PrüferInnen für das TKM-Ausbildungs-Zertifikat (siehe 5). TKM-Basisseminare zur Erlangung des TKM-Zertifikats werden von TKM-AusbilderInnen bzw. den in Tab. 2 genannten Berufsgruppen durchgeführt. Die konkrete Umsetzung der Konzeption von TKM-Basisseminaren (Inhalte, Ablauf, ReferentInnen, Prüfungsmodalitäten) muss von der Deutschen interdisziplinären Gesellschaft für Dysphagie (DGD) akkreditiert werden.

Die Prüfung der Eingangsvoraussetzungen (siehe 3.1, 3.2, 4.1), Prüfungszulassungen und das Ausstellen der Zertifikate nach erfolgreich abgeschlossener Ausbildung werden bei der DGD beantragt, dazu sind die erforderlichen Nachweise schriftlich über die Webseite der DGD (www.dg-dysphagie.de) einzureichen. Bestätigungen können durch Vorgesetzte (logopädische Praxis‑, Team- oder Fachleitungen oder ärztliche Vorgesetzte) ausgestellt werden. Im außerklinischen oder ambulanten Sektor tätige LogaS können die Eingangsvoraussetzungen durch überweisende ÄrztInnen bestätigen lassen oder den Nachweis durch Einreichen einer entsprechenden Anzahl von Verordnungen mit dem Diagnoseschlüssel R 13.1 erbringen.

## 8. Formale Aspekte und Delegation

### 8.1. Delegation

Die Zusammenarbeit von ÄrztInnen und LogaS wird unter dem Begriff der „Delegation ärztlicher Tätigkeit“ geregelt. Delegation meint die Übertragung ärztlicher Aufgaben auf nichtärztliches Personal und ist an bestimmte Voraussetzungen geknüpft. Generelle Kriterien für die Delegationsfähigkeit gibt es jedoch nicht. Allgemein hängt die Übertragbarkeit ärztlicher Aufgaben ab von der Schwere des Eingriffs (Schwierigkeit), der Häufigkeit und Beherrschbarkeit möglicherweise auftretender Komplikationen (Risiko) und der Schwierigkeit der angewandten Technik (Beherrschbarkeit). Die Übertragung einer Aufgabe setzt voraus, dass ein persönliches Tätigwerden von ÄrztInnen nach Art und Schwere des Krankheitsbildes oder des Eingriffs nicht erforderlich ist und DelegationsadressatInnen die erforderliche Qualifikation, Zuverlässigkeit und Erfahrung aufweisen [22].

Delegationsentscheidungen „*muss der Arzt von der Qualifikation des jeweiligen Mitarbeiters abhängig machen*“ (Bundesärztekammer und Kassenärztliche Bundesvereinigung, 2008, A 2174 [23]). „*Je besser das Personal qualifiziert ist, desto mehr Leistungen können auch delegiert werden*“ [24, S. 15].

Aus juristischer Sicht wird betont, dass delegationsfähige Aufgaben nicht durch gesetzliche Regelungen, sondern grundsätzlich von der Medizin selbst festzulegen sind und die beteiligten Berufsgruppen die Abgrenzung zwischen ärztlichen und nichtärztlichen Leistungen selbst vorzunehmen haben. Die aktuellen Abgrenzungen sind dabei nicht starr, sondern historisch gewachsen und unterliegen national und international dem Wandel [25, 26]. Als nicht delegationsfähig werden jedoch sogenannte höchstpersönliche Leistungen der Ärztin/des Arztes erachtet, welche dem Kernbereich ärztlicher Tätigkeit zuzuordnen sind. Dazu gehören u. a. Anamnese, Indikationsstellung, Aufklärung und Beratung der PatientInnen [23, 26].

### 8.2. Pflichten der delegierenden ÄrztInnen und der DelegationsadressatInnen

DelegationsadressatInnen sind in diesem Zusammenhang die ausführenden TherapeutInnen. „Bei ihnen sind die Ebenen der formalen und der materiellen Qualifikation zu unterscheiden: Die formale Qualifikation ist die durch ein Ausbildungszeugnis bescheinigte Fähigkeit […]. Unter der materiellen Qualifikation ist demgegenüber die tatsächliche Befähigung der Delegationsadressat*innen zur Durchführung der angewiesenen Maßnahme zu verstehen“ [22, S. 227].

Delegation bedeutet die Entlastung von ÄrztInnen sowie die Aufwertung der nichtärztlichen BerufsträgerInnen und ist deshalb im Grundsatz positiv zu bewerten [24]. DelegationsadressatInnen dürfen jedoch nur Aufgaben übernehmen, die ihrer formalen und materiellen Qualifikation entsprechen. Sollte eine Aufgabe übernommen werden, die die persönlichen Fähigkeiten und Möglichkeiten übersteigt und nicht dem Standard der jeweiligen Berufsgruppe genügt, stellt dies einen Qualitätsmangel (§ 276 BGB) dar, der zum Schadensersatz verpflichtet; es kommt zu einer Haftung aus Übernahmeverschulden. Die Rechtsprechung hat zudem mehrfach entschieden, dass die Übertragung und Ausführung von Aufgaben an nicht hinreichend qualifizierte Personen einen Behandlungsfehler darstellt [26].

Soll eine Leistung delegiert werden, so muss folglich die formale Qualifikation der Mitarbeiterin/des Mitarbeiters festgestellt werden. Dies ist die abgeschlossene und befähigende Ausbildung in einem Fachberuf des Gesundheitswesens (hier: Logopädie oder akademische Sprachtherapie), das TKM-Zertifikat bzw. TKM-Ausbildungs-Zertifikat zeigt den Qualifikationsnachweis im Bereich des TKM an. Ferner müssen sich die Delegierenden zu Beginn der Zusammenarbeit davon überzeugen, dass die Leistungen der Mitarbeiterin/des Mitarbeiters auch tatsächlich den qualitativen Anforderungen entsprechen. Die Qualität der erbrachten Leistungen (materielle Qualifikation) ist stichprobenartig zu überprüfen [23].

### 8.3. Delegationsform

„*Eine ärztliche Delegation bedarf aus Rechtsgründen nicht der Schriftform. […] Das ärztliche Berufsrecht und die ärztliche Sorgfaltspflicht verlangen lediglich, dass die Anordnung klar und eindeutig erfolgt und die Übertragung der Leistung zulässig ist*“ [26, S. 7]. Die Dokumentation inhaltlicher Festlegungen zwischen den Delegierenden und den DelegationsadressatInnen wird dennoch zu Beweiszwecken empfohlen.

### 8.4. Delegation im TK-Management

Im klinischen, außerklinischen und ambulanten Alltag ist das TKM bereits überwiegend delegiert. DelegationsadressatInnen sind Intensivpflegekräfte, PflegetherapeutInnen und, je nach Ausbildungsstand, LogaS. In einer Studie des Deutschen Krankenhausinstituts werden das endotracheale Absaugen wie auch das Anlegen einer transnasalen Sonde als delegierbare Leistungen an nichtärztliches Personal nach qualifizierender Ausbildung und spezifischer Schulung gelistet [26]. Weitere therapeutische Maßnahmen (z. B. Cuff-Entblockung, Okklusionstraining ggf. unter Leckage-Beatmung, Anpassung des Trachealkanülentyps, Färbetests, Möglichkeiten und Kontraindikationen oraler Ernährung mit TK, Sekretmanagement) sind nur im interdisziplinären Austausch sinnvoll festzulegen und erfordern Absprachen zwischen den Delegierenden, den DelegationsadressatInnen und weiteren Disziplinen. Der Einsatz von Medikamenten, Inhalativa und Lokalanästhetika im Rahmen des TKM erfordert eine ärztliche Verordnung.

Im Gegensatz zur Klinik ist in der außerklinischen Intensivpflege (AKI) und in der ambulanten logopädisch-sprachtherapeutischen Versorgung die Anwesenheit delegierender ÄrztInnen häufig nicht gegeben. Nachdem in diesen Settings kein akutmedizinischer Behandlungsbedarf besteht und die medizinische Überwachung in der AKI durch die Intensivpflege durchgehend gesichert ist, kann davon ausgegangen werden, dass PatientInnen in der AKI aus medizinischer Sicht zur Durchführung des TKM im Rahmen der Dysphagietherapie geeignet sind. Im Sinne eines adäquaten Risikomanagements muss aber die Notwendigkeit inhaltlicher Absprachen (Festlegung therapeutischer Maßnahmen, Zielvereinbarungen) und gegenseitiger Information (Risikobewertungen, Zustandsveränderungen wie z. B. bronchopulmonale/internistische Komplikationen, Änderungen der Beatmungszeiten oder -modi) betont werden. § 2 (4) der AKI-Richtlinie des G‑BA ist zu beachten.

Die AutorInnen und die beteiligten Fachgesellschaften sind der Ansicht, dass die im TKM-Curriculum vermittelten Tätigkeiten des TKM in der Dysphagietherapie in Schwierigkeit, Risiko und Beherrschbarkeit an LogaS in der Klinik, der AKI und in der Ambulanz delegierbar sind und ein persönliches Tätigwerden von ÄrztInnen, was die oben näher ausgeführten delegierbaren Leistungen betrifft, nicht zwingend erfordern. Regelhafter interdisziplinärer Austausch mit dem Ziel inhaltlicher Absprachen und Festlegungen ist notwendiger Bestandteil der Delegation. Die formale Qualifikation wird von LogaS im Rahmen des vorgestellten TKM-Curriculums erworben, die materielle Qualifikation ist im Einzelfall durch die delegierenden Ärzte zu prüfen. Die Delegation des TKM in der Dysphagietherapie an entsprechend qualifizierte LogaS wird im Sinne einer ausreichenden und verbesserten Versorgung tracheotomierter PatientInnen mit Dysphagie unter den genannten Voraussetzungen empfohlen.

## Fazit für die Praxis

Das TKM-Curriculum wurde von medizinischen und therapeutischen Fachgesellschaften entwickelt und konsentiert. Es beschreibt formale Qualifikationsstufen und definiert Tätigkeitsbereiche in der Versorgung tracheotomierter PatientInnen durch LogopädInnen und akademische SprachtherapeutInnen. Das TKM-Curriculum stellt damit einen wesentlichen Baustein zur Sicherstellung und Verbesserung der Versorgung tracheotomierter PatientInnen in der Klinik, der Ambulanz und der außerklinischen Intensivpflege dar und ist eine Grundlage für die Delegation von Leistungen im Trachealkanülenmanagement.
